# Pre-hospital delay in patients with first time myocardial infarction: an observational study in a northern Swedish population

**DOI:** 10.1186/s12872-016-0271-x

**Published:** 2016-05-12

**Authors:** Gunnar Nilsson, Thomas Mooe, Lars Söderström, Eva Samuelsson

**Affiliations:** Department of Public Health and Clinical Medicine, Unit of Research, Education and Development - Östersund, Umeå University, Umeå, Sweden; Department of Public Health and Clinical Medicine, Umeå University, Umeå, Sweden; Unit of Research, Education and Development, Östersund Hospital, Region Jämtland Härjedalen, Östersund, Sweden

**Keywords:** Myocardial infarction, Observational study, Pre-hospital delay, Primary care

## Abstract

**Background:**

In myocardial infarction (MI), pre-hospital delay is associated with increased mortality and decreased possibility of revascularisation. We assessed pre-hospital delay in patients with first time MI in a northern Swedish population and identified determinants of a pre-hospital delay ≥ 2 h.

**Methods:**

A total of 89 women (mean age 72.6 years) and 176 men (mean age 65.8 years) from a secondary prevention study were enrolled in an observational study after first time MI between November 2009 and March 2012. Total pre-hospital delay was defined as the time from the onset of symptoms suggestive of MI to admission to the hospital. Decision time was defined as the time from the onset of symptoms until the call to Emergency Medical Services (EMS). The time of symptom onset was assessed during the episode of care, and the time of call to EMS and admission to the hospital was based on recorded data. The first medical contact was determined from a mailed questionnaire. Determinants associated with pre-hospital delay ≥ 2 h were identified by multivariable logistic regression.

**Results:**

The median total pre-hospital delay was 5.1 h (IQR 18.1), decision time 3.1 h (IQR 10.4), and transport time 1.2 h (IQR 1.0). The first medical contact was to primary care in 52.3 % of cases (22.3 % as a visit to a general practitioner and 30 % by telephone counselling), 37.3 % called the EMS, and 10.4 % self-referred to the hospital. Determinants of a pre-hospital delay ≥ 2 h were a visit to a general practitioner (OR 10.77, 95 % CI 2.39–48.59), call to primary care telephone counselling (OR 3.82, 95 % CI 1.68–8.68), chest pain as the predominant presenting symptom (OR 0.24, 95 % CI 0.08–0.77), and distance from the hospital (OR 1.03, 95 % CI 1.02–1.04). Among patients with primary care as the first medical contact, 67.0 % had a decision time ≥ 2 h, compared to 44.7 % of patients who called EMS or self-referred (*p* = 0.002).

**Conclusions:**

Pre-hospital delay in patients with first time MI is prolonged considerably, particularly when primary care is the first medical contact. Actions to shorten decision time and increase the use of EMS are still necessary.

**Electronic supplementary material:**

The online version of this article (doi:10.1186/s12872-016-0271-x) contains supplementary material, which is available to authorized users.

## Background

Pre-hospital delay in myocardial infarction (MI) is associated with increased mortality [1, 2] and decreased possibility of revascularisation [3, 4]. Delay times exceeding 2.0 h are still commonly reported [5–8]. A cut-off time for pre-hospital delay is arbitrary, as mortality increases with time to reperfusion therapy [1, 9]. However, a 2-h cut-off is often applied because MI patients treated within 2 h receive the most clinical benefit from reperfusion therapy [3, 10].

Total pre-hospital delay can be divided into decision time (time from the onset of symptoms suggestive of MI until the call for medical help) and transport time (time from the call for medical help to hospital admission), also called “home-to-hospital delay” [11], with the decision time as the major part [12–15]. The scientific terminology for pre-hospital delay is not consistent; “time-to-treatment” and “treatment-seeking delay” are alternative terms, making comparisons between studies difficult [16].

Several determinants are associated with pre-hospital delay, including low socio-economic status, female gender, co-morbidities (e.g., diabetes and coronary disease), the patient’s cognitive and emotional status, and determinants related to the healthcare provider [17, 18]. In some reports, patients with primary care as the first medical contact (FMC) have an increased pre-hospital delay [7, 19–21], often with less severe cardiac events than other patients [22]. Primary care clinics and telephone counselling services are frequently the FMC for patients with a suspected MI [7, 21], as symptoms related to MI often are not identified as cardiac [23]. Symptoms of MI may also be vague or atypical, leading to delayed care [24–27]. The impact of a previous MI on pre-hospital delay has varied in different studies. Results have shown shorter [28–31], longer [2, 32], or even neutral [21, 33] pre-hospital delays in association with a previous MI.

Pre-hospital delay in MI is related to the context [22, 27], and research on this issue should be based on data-sets that include relevant socio-demographic and healthcare-related data. The northern Swedish setting is characterised by long distances to the hospital, an aged population, and low to average education level. Traditionally, primary care has been the FMC for both acute and chronic diseases. By combining data from three different sources, we provide a more detailed picture of the pre-hospital delay issue compared to studies using a narrower data catch. Our aim was to assess the pre-hospital delay in men and women in a northern Swedish population with first time MI, and to identify determinants of a prolonged pre-hospital delay ≥ 2 h.

## Methods

### Participants

Participants in this observational study were recruited from the population of Region Jämtland Härjedalen, northern Sweden (in 2012: population 126 201, 53 % living in rural communities and 47 % in the capital community) [34]. The capital community of the region, Östersund, is the location of the regional hospital with clinics for cardiology and emergency medical services (EMS). The distance from participants’ place of residence to the hospital ranged from 0.4 to 234 km. A referral from a GP was not required for patients to access emergency care or ambulance transport to a hospital in cases of chest pain suggestive of myocardial infarction. The primary care clinics were run by the regional healthcare authorities or contracted to provide primary care on the same taxation system and with the same patient charges. Participating patients were hospitalised with MI type 1, according to the universal definition [35], between November 26, 2009 and March 26, 2012. Eligible participants were identified from a population-based secondary prevention study that recruited patients after acute coronary syndromes (ACS) and stroke, within the Region Jämtland Härjedalen [36]. For patients living in rural communities, ambulance services and primary care clinics were accessible locally. Medical telephone counselling was available from primary care clinics 08:00 a.m. - 17:00 p.m. on weekdays and from Swedish Healthcare Direct (SHD) at all hours, with the possibility of directing patients to the Emergency Medical Services (EMS) or a primary care clinic as appropriate. The SHD, a part of the primary care organisation of the region, provided medical telephone counselling by nursing staff as a complementary service to the primary care clinics. The EMS alarm number was also accessible for calls from the public on a 24-h basis. The EMS with ambulance-based pre-hospital care, including thrombolytic therapy, was organised by the Emergency Care Centre, Östersund Hospital. Visits to primary care, emergency care, and ambulance transport were subject to patient charges of approximately 15–27 € during the study period. Deceased patients and patients declining consent or with insufficient data on pre-hospital delay were excluded from the present study.

### Data sources and measurements

We used three different data sources. First, to acquire demographic and medical baseline data, a structured interview was carried out during the initial hospitalisation by nursing staff engaged in the secondary prevention study. The outline of the secondary prevention study of patients with ACS was published previously [36]. Second, previous chest pain symptoms, expectations of medical care, pre-hospital events, and FMC before admission to the hospital were recorded from a postal questionnaire sent to patients within 3–6 months after MI. Two reminders were sent to ensure participation. Third, for patients transported by ambulance, the symptoms reported by the patient at triage, the time of call to the EMS, and the time of admission to the hospital were recorded from ambulance records. For patients with private transport to the hospital, triage data and time of admission to the hospital were recorded from prospective records at the Emergency Care Centre.

**Fig. 1:**
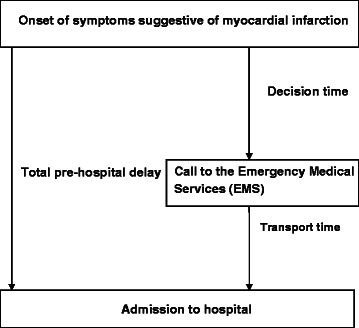
Pre-hospital delay and definition of time intervals

Time of onset of symptoms suggestive of MI was determined during the episode of care, by nursing staff engaged in the secondary care study. Uncertainty in the time of symptom onset was estimated in hours, more or less, relative to the recorded onset time. The definitions of time intervals are explained in Fig. 1. For patients with private transport, only total pre-hospital delay was possible to calculate because the time to call to the EMS was unavailable.

Patients’ expectations for medical care the day of admission to the hospital was assessed on a visual analogue scale from 0–100. The pain intensity at triage was assessed on a visual analogue scale from 0–10. If several assessments of pain were recorded during triage, the highest value was chosen. If a statement of no pain was recorded, the value was recorded as 0.

The distance from the patient’s residence to the hospital was computed by Google Maps. Socio-economic classification was based on the Swedish Socioeconomic Classification (SEI) [37]. Marital status was determined from hospital records.

Three questions on previous chest pain symptoms were originally used in the “Rose angina questionnaire” [38–40] and the Swedish translation for primary care patients assessed for coronary disease [41]. Questions on the sequence of events before admission to the hospital were presented with fixed alternatives. The question “In your own opinion, did you suspect a myocardial infarction the day you fell ill?” was asked with yes and no as potential answers. A question on FMC before admission was presented with fixed alternatives, with the possibility of providing additional information, for classification into the following categories: “Personal visit to a GP before referral”; “Referral by call to a primary care centre/Swedish Healthcare Direct”; “Called the Emergency Medical Services;” and “Self-referred to hospital”. Ambulance transport of patients was confirmed by ambulance records stating the location, date, time, medical actions, and condition of the patient at triage. For patients with private transport, the same triaging procedure was carried out at the emergency department. Presenting symptoms were classified as: “Predominantly chest pain symptoms”, e.g., pain, ache, burn, or pressure in the chest; “Predominantly other pain symptoms”, e.g., predominance of pain in the abdomen, arm, shoulder, or neck; and “Predominance of symptoms other than pain”, e.g., severe fatigue, syncope, or circulatory shock.

MIs were diagnosed in accordance with the universal definition of MI type 1 [35]. The type of MI, ST elevation MI (STEMI) or non-ST elevation MI (NSTEMI), was not treated as a determinant of pre-hospital delay because it is an outcome measurement from the pre-hospital perspective.

### Delay caused by medical misjudgement

All medical records were scrutinised for patients who were sent home from clinics or kept waiting to detect cases in which medical misjudgement contributed to a pre-hospital delay ≥ 2 h.

### Statistical analysis

Patient characteristics are presented as proportions, means, or medians. The median and inter quartile range (IQR) were used for highly skewed distributions. To compare proportions, we used the chi-squared test or Fisher’s exact test as appropriate. To compare means or medians, we used the Student’s *t*-test (two sided) or the Mann–Whitney *U*-test as appropriate. We used univariate logistic regression to identify determinants of pre-hospital delay and *p* < 0.25 for determinants to be included in the multivariable logistic model. We reduced the model stepwise by excluding the least significant variable manually until only significant variables remained. The level of significance was set at *p* < 0.05. To assess the discriminatory power of the multivariable model, we used receiver operating characteristic (ROC) curves and calculated the area under the curve (AUC) [42, 43]. Statistical analyses were performed in the software IBM SPSS version 22.

## Results

### Descriptive data

**Fig. 2:**
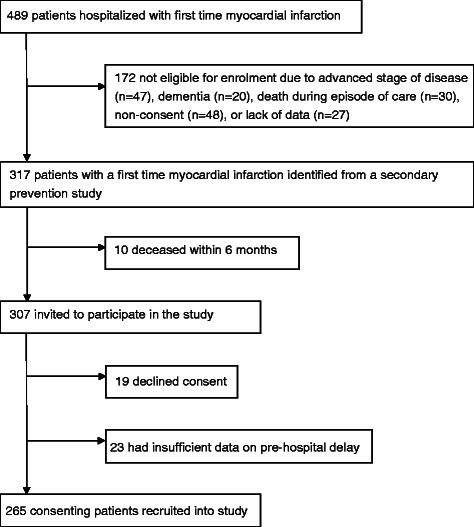
Participant recruitment

We recruited 265 consenting patients, 89 of which were women, to take part in this study (Fig. 2). The mean patient age was 68.1 years; the mean age of participating women was 72.6 years. “Manual workers” was the predominant socio-economic group (62.7 %). The receiving hospital for 258 patients was the central hospital in Östersund; the other seven patients were admitted to other Swedish hospitals due to temporary visits outside their normal place of residence. The FMC was primary care in 52.3 % of all cases (22.3 % as a visit to a general practitioner (GP) and 30 % by telephone counselling), 37.3 % called the EMS, and 10.4 % self-referred to the hospital. A majority of patients (76.6 %) used ambulance transport (198 by road and 5 by air ambulance). Patients visiting a GP, calling a primary care clinic/SHD, or calling the EMS as the FMC were transported by ambulance in 72.4, 79.5, and 99 % of cases, respectively. Finally, 97 patients (36.6 %) were diagnosed as STEMI (21 women and 76 men), and the others as NSTEMI.

### Main results

The median total pre-hospital delay was 5.1 (IQR 18.1) hours, with a median decision time of 3.1 (IQR 10.4) hours and median transport time of 1.2 (IQR 1.0) hours. No differences were found between men and women (Table 1). The median transport time was 0.78 (IQR 0.5) hours in the central community and 1.65 (IQR 1.1) hours in the rural communities (*p* <0.001). No significant differences were found in the decision time or total pre-hospital delay between the central and rural communities. The uncertainty of the time of symptom onset was a median 0.0 h (IQR 1.0), with the 80th percentile at 2.0 h.

**Table 1:** Total pre-hospital delay, decision time, and transport time for patients with first time myocardial infarction

Characteristic	Total	Men	Women	*P*-value, Mann–Whitney *U* Test
	*n* = 265	*n* = 176 (66.4 %)	*n* = 89 (33.6 %)	
Total pre-hospital delay, median (IQR)	5.1 (18.1)	5.9 (17.4)	4.1 (19.3)	0.436
Decision time, median (IQR)^a^	3.1 (10.4)	3.7 (10.1)	2.0 (11.8)	0.411
Transport time, median (IQR)^a^	1.2 (1.0)	1.3 (1.0)	1.2 (1.0)	0.052

All data are given in hours. Total pre-hospital delay is the time between onset of symptoms suggestive of myocardial infarction and admission to the hospital. Decision time is the time between onset of symptoms suggestive of myocardial infarction and the call to Emergency Medical Services. Transport time is the time between the call to Emergency Medical Services and admission to the hospital. ^a^Calculated for 200/203 patients transported to the hospital by ambulance

A highly skewed distribution in total pre-hospital delay and decision time was observed with wide IQRs among both men and women (Table 1 and Additional file 1). Patients with a total pre-hospital delay ≥2 h lived farther from the hospital and were more likely to have consulted a GP before admission to the hospital, to be diabetic, and to report recurrent angina symptoms than those with a total pre-hospital delay <2 h. Patients with a total pre-hospital delay <2 h were more likely to have called the EMS or self-referred to the hospital, and they were more likely to report chest pain as the predominant symptom at triage than those with a total pre-hospital delay ≥2 h (Table 2).

**Table 2:** Characteristics of first time myocardial infarction patients according to total pre-hospital delay (*n* = 265)

Characteristic	<2 h	≥2 h	*P*-value
	*n* = 52 (19.6 %)	*n* = 213 (80.4 %)	
Mean age, years (SD)	67.5 (11.3)	68.2 (11.7)	0.668
Female sex	19/52 (36.5 %)	70/213 (32.9 %)	0.615
Married or cohabiting	23/32 (71.9 %)	106/172 (61.6 %)	0.270
Distance to hospital in km, median (IQR)	7.0 (27.5)	49.8 (87.4)	<0.001
University or college degree	7/51 (13.7 %)	31/213 (14.6 %)	0.880
Manual worker	33/51 (66.0 %)	132/212 (61.9 %)	0.746
Medical history			
Previous revascularisation	2/52 (3.8 %)	7/213 (3.3 %)	0.691
Previous stroke or TIA	3/52 (5.8 %)	10/213 (4.7 %)	0.742
Angina pectoris, current diagnosis	2/52 (3.8 %)	20/213 (9.4 %)	0.267
Hypertension, current diagnosis	25/52 48.1 %)	104/213 (48.8 %)	0.923
Diabetes mellitus, medication for	5/52 (9.6 %)	46/213 (21.6 %)	0.049
Dyslipidaemia, medication for	6/52 (11.5 %)	44/213 (20.7 %)	0.132
Previous chest pain symptoms			
Ever have chest pain or discomfort in the chest	21/52 (40.4 %)	63/203 (31.0 %)	0.201
Chest pain walking at an ordinary pace on the level	3/51 (5.9 %)	23/198 (11.6 %)	0.232
Chest pain walking uphill or in a hurry	14/51 (27.5 %)	68/202 (33.7 %)	0.397
On the day of admission to hospital			
Myocardial infarction suspected by patient	23/52 (44.2 %)	71/210 (33.8 %)	0.161
“I decided by myself to seek medical care”	23/49 (46.9 %)	99/209 (47.47.4 %)	0.957
“I took advice from a next of kin/friend”	9/49 (18.4 %)	50/209 (23.9 %)	0.405
“A next of kin/friend contacted medical care”	17/49 (34.7 %)	60/209 (28.7 %)	0.410
Expectations of medical care, mean (SD)^a^	80.2 (15.0)	76.2 (21.4)	0.208
First medical contact before admission to hospital			
Personal visit to a GP before referral	2/52 (3.8 %)	56/208 (26.9 %)	<0.001
Referred by call to a primary care centre/Swedish Healthcare Direct	10/52 (19.2 %)	68/208 (32.7 %)	0.058
Called the Emergency Medical Services	27/52 (51.9 %)	70/208 (33.7 %)	0.015
Self-referred to hospital	13/52 (25.0 %)	14/208 (6.7 %)	<0.001
Ambulance transport to hospital	36/52 (69.2 %)	167/213 (78.4 %)	0.161
Symptoms reported by patient at triage			
Predominantly chest pain	48/52 (92.3 %)	170/211 (80.6 %)	0.044
Predominantly other pain	3/52 (5.8 %)	27/211 (12.8 %)	0.153
Symptoms other than pain predominated	1/52 (1.9 %)	14/211 (6.6 %)	0.189
Symptom onset related to physical strain	10/52 (19.2 %)	37/210 (17.6 %)	0.786
Recurrent angina within 2 weeks before admission	9/52 (17.3 %)	69/213 (32.4 %)	0.032
Pain intensity, mean (SD)^b^	6.7 (2.7)	5.5 (3.1)	0.099

*TIA* transitory ischaemic attack^a^Visual analogue scale from 0 to 100 where 0 is lowest and 100 is highest possible expectations; five patients with missing values^b^Visual analogue scale from 0 to 10 where 0 is no pain and 10 is worst possible pain; assessed at triage in 115/265 patients

Characteristics associated with a total pre-hospital delay ≥ 2 h in the adjusted model were: visit to a GP before referral (OR 10.77, 95 % CI 2.39–48.59), referral by a call to a primary care centre/SHD (OR 3.82, 95 % CI 1.68–8.68), chest pain as the predominant symptom at triage (negative association; OR 0.24, 95 % CI 0.08–0.77), and distance (km) to hospital (OR 1.03, 95 % CI 1.02–1.04) (Table 3). Patients in contact with primary care, as a GP visit or by telephone counselling, also had a prolonged decision time (Table 3). Chest pain as the predominant symptom at triage was associated with a shorter decision time (Table 3). We examined the characteristics of the regression model for interaction with gender, but none of the findings were significant. Age, gender, and levels of scholarship did not contribute to significant improvement of the multivariable model. The discriminatory ability of the multivariable model (Table 3) was evaluated by ROC curves; the AUC was 0.84 (95 % CI 0.79–0.90, *p* <0.001) for total pre-hospital delay and 0.68 (95 % CI 0.60–0.76, *p* <0.001) for decision time.

**Table 3:** Characteristics associated with prolonged pre-hospital delay in patients with first time myocardial infarction

Characteristic	Total prehospital delay ≥ 2 h		Decision time ≥ 2 h	
	Adjusted OR (95 % CI)	*P*-value	Adjusted OR (95 % CI)	*P*-value
Personal visit to a GP before referral	10.77 (2.39–48.59)	0.002	3.85 (1.66–8.90)	0.002
Referred by call to a primary care centre/Swedish Healthcare Direct	3.82 (1.68–8.68)	0.001	2.00 (1.03–3.87)	0.041
Chest pain predominating symptom at triage	0.24 (0.08–0.77)	0.016	0.34 (0.12–0.90)	0.031
Distance to hospital, km	1.03 (1.02–1.04)	<0.001	1.00 (1.00–1.01)	0.586

Total pre-hospital delay is the time between onset of symptoms suggestive of myocardial infarction and admission to the hospital. Decision time is the time between onset of symptoms suggestive of myocardial infarction and the call to Emergency Medical Services

Among patients with primary care as the FMC, 67.0 % had a decision time ≥ 2 h, compared to 44.7 % of patients calling the EMS or self-referring to the hospital (*p* = 0.002). Compared to patients who self-referred or called the EMS before admission to the hospital, primary care patients were younger (mean age 66.3 years (SD 12.0) vs. 69.7 (SD 10.9) years, *p* = 0.016) and lived a greater distance from the hospital (median distance 37.5 km (IQR 10.0–99.5) vs. 21.0 km (IQR 4.0–68.5), *p* = 0.012). Patients with a FMC to primary care more often reported recurrent angina symptoms preceding the MI (34.6 % vs. 21.8 %, *p* = 0.022), a lower pain intensity at triage (5.1 (SD 3.1) vs. 6.3 (SD 2.9), *p* = 0.030), and less frequently asked a friend/next of kin for help before admission to the hospital (20.5 % vs. 38.8 %, *p* = 0.001) than patients who self-referred or called the EMS. Patients with a FMC to primary care were less frequently diagnosed with STEMI than patients who self-referred or called the EMS as the FMC (25.7 % vs. 49.2 %, *p* < 0.001). Among primary care patients with private transport to the hospital and lived in rural communities, the total pre-hospital delay time increased stepwise compared to the total population (Table 4).

**Table 4:** Total pre-hospital delay according to first medical contact (FMC), transport mode, and residency

Characteristic	Number of patients	Total pre-hospital delay in hours, median (IQR)
Total	265	5.1 (18.1)
Primary care as FMC	136	8.7 (33.3)
Primary care as FMC and private transport to hospital	32	20.9 (69.1)
Primary care as FMC, private transport to hospital, and rural residency	12	74.0 (140.8)

Primary care as FMC: Visit to a GP, call to a primary care centre or Swedish Healthcare Direct

### Delay caused by medical misjudgement

We identified three patients with delayed care ≥ 2 h due to medical misjudgement; two were sent home from primary care clinics but returned within 12 to 24 h, and one patient was delayed for 2 h at a primary care centre before referral.

### Non-participants

Non-participating patients did not differ significantly with respect to age, gender, or distance from the hospital compared to participants.

## Discussion

### Key findings

The median total pre-hospital delay was 5.1 h, with decision time as the major contributor. The FMC was to primary care (as a GP visit or by telephone counselling) in approximately half of all patients. Visiting a GP or calling primary care for telephone counselling prior to hospital admission were both associated with a total pre-hospital delay and decision time ≥ 2 h. Chest pain as the predominant symptom at triage was associated with shorter total pre-hospital delay and decision time.

### Interpretation of findings

The pre-hospital delay among our study participants exceeded that reported in several previous studies on ACS and MI. Pre-hospital delay in the European patients’ study arm of the Global Registry of Acute Coronary Events (GRACE) study was a median 2.3–2.7 h for STEMI and 2.7–3.1 h for NSTEMI cases between 2000 and 2006 (lowest value in 2006) [28]. In the Northern Sweden MONICA Study, a delay time ≥ 2 h was found in 64 % of patients with diabetes and 58 % of non-diabetics [44]. In a cohort of Norwegian patients with first time MI, 52 % of women and 51 % of men had a total pre-hospital delay exceeding 2 h [19]. A pre-hospital delay similar to our data was reported in an Irish setting in 2013; the median pre-hospital delay for STEMI patients was 2.7 h and for NSTEMI patients 4.5 h [7].

There are several possible explanations for the prolonged delay in our study. First, unlike the MONICA cohort and the Norwegian multicentre study by Lovlien et al [19, 44], we did not apply an upper age limit, and we recruited a somewhat higher proportion of female participants. Second, the prolonged pre-hospital delay among our study patients is likely related to the context of medical care, as primary care as the FMC is recommended for patients and the EMS is the second choice in most circumstances [7, 19–22]. In our study, primary care as the FMC was associated with both prolonged pre-hospital delay and decision time. Pre-hospital delay attributable to healthcare provider contact was described previously [22, 30, 45] but remains to be addressed because such provider-related delay may account for more loss in total delay time compared to patient delay [46–48]. This problem is even more important if patients believe that calling primary care, and not EMS, is always the appropriate action [49]. This could explain the association between primary care as the FMC and pre-hospital delay that we observed.

Furthermore, many of our study participants lived far from the hospital, with consequences on transport time. However, 76.6 % used an ambulance for transportation to the hospital, which is a greater proportion of patients than in previous reports from Ireland, Australia, and Sweden (40–50 % of ACS patients with ambulance transport) [50–52]. Among our patients, 37.3 % primarily called the EMS, 99 % of which were transported by ambulance, compared to 72.4 % of patients who visited a GP as the FMC. GP visits before hospital admission may have resulted in delayed transport and fewer patients transported by ambulance, as recently reported in an Irish setting [51]. Ambulance stations with access to thrombolytic therapy were located in all rural communities, and a shortage of ambulance resources is unlikely to have contributed to delayed care among our patients.

In our study, the average education level was low and a majority of participants were manual workers, but these were not determinants of pre-hospital delay. Previous research has demonstrated a relationship between low socio-economic level and pre-hospital delay [18, 53, 54], but definitions of socio-economic status and cut-offs for pre-hospital delay differ, making comparisons difficult.

### Future aspects

The association between the patient’s choice of FMC and pre-hospital delay force us to rethink the kind of assessment of chest pain that is most appropriate in primary care. Clinical prediction rules to rule out coronary disease in low-risk patients has been proposed as one possibility for selecting patients with chest pain for cardiologic care [55, 56]; point-of-care troponin T testing is another possibility [57], but such measures are unlikely to decrease pre-hospital delay related to health care providers. As proposed previously, medical telephone counselling should be the focus of an epidemiological study to further develop the management of ACS calls [22]. Our findings support patients with new onset chest pain being encouraged to call the EMS and not primary care telephone counselling. As public campaigns to reduce pre-hospital delay have yielded negative results [58], future efforts should target high-risk patients, preferably by individualised patient education, which has been reported to reduce pre-hospital time in patients with recurrent ACS episodes [15]. A high-risk approach is further supported by the increasing delay-time among certain subgroups in our study, such as primary care patients with private transport to the hospital and rural residency (Table 4). The discriminatory ability of the multivariable model supports the core determinants of pre-hospital delay being related to a patient’s decision-making process and choice of health care provider [22, 46].

### Strengths and limitations

We used different data sources to provide detailed information on each study participant. A high participation rate (86.3 %) and the population-based study approach strengthened the external validity. Data on symptom presentation at triage were recorded from ambulance and emergency care records, reflecting conditions at the time of care as closely as possible. Previous medical history, socio-economic status, and time of symptom onset were assessed by trained nursing staff during the episode of care. The exact time of the onset of symptoms indicating MI can be hard to establish and is a common problem in studies reporting on pre-hospital delay [16]. An estimation of the uncertainty in the time of symptom onset was included in our study plan.

The subdivision of total pre-hospital delay into decision time and transport time was not possible to calculate in patients with private transport to the hospital, which limits our analysis of decision time and transport time to patients with ambulance transport. Delay due to medical misjudgement was determined from retrospective data, which may have been insufficient. We recruited patients from a population-based secondary prevention program after MI and ACS, meaning that the generalisability of our findings is limited to surviving patients eligible for a prevention programme. The rural context, with many patients living distantly from hospital but with access to primary care and ambulance services, is another limitation for the overall generalisability of our findings. The postal questionnaire covering previous chest pain symptoms, sequence of events, and FMC prior to hospitalisation was delivered within 3-6 months after the MI, and recall bias cannot be ruled out.

Comparisons across different studies are complicated by different definitions of pre-hospital delay [16]. We applied a bivariate approach with a 2-h cut-off to identify determinants associated with pre-hospital delay. This time limit was chosen from a theoretical point of view [3] and to allow comparisons with other studies using the same cut-off [14, 19, 28, 44, 59].

## Conclusions

In this study of patients in a northern Swedish population with first time MI, the total pre-hospital delay was considerably prolonged (median 5.1 h), with decision time as the major contributor (median 3.1 h). Primary care patients had a longer pre-hospital delay, mainly due to a longer decision time. Actions to shorten decision time and increase the use of EMS are still necessary.

### Ethics approval and consent to participate

The study was approved by the Regional Ethics Review Board, Umeå University (reference number Dnr 09–133 M and 2010/302–32 M). All participants provided written informed consent.

### Consent for publication

Not applicable.

### Availability of data and materials

Patient level data will be available on request, provided that an approval is given from the Regional Ethics Review Board at Umeå University, Sweden.

## Additional file

Additional file 1: Box plots of total pre-hospital delay and decision time. (PDF 171 kb)

## Abbreviations

ACS: Acute coronary syndrome
AUC: Area under the curve
ECG: Electrocardiogram
EMS: Emergency medical services
FMC: First medical contact
GP: General Practitioner
MI: Myocardial infarction
NSTEMI: Non-ST elevation myocardial infarction
ROC: Receiver operating characteristic
SHD: Swedish Healthcare Direct
STEMI: ST elevation myocardial infarction
