# Supplementary figures and images for: Pre-hospital delay in patients with first time myocardial infarction: an observational study in a northern Swedish population
Source: BMC Cardiovasc Disord. 2016 May 12;16:93. doi: 10.1186/s12872-016-0271-x (PMC4866271; doi:10.1186/s12872-016-0271-x)

**Additional data, file 1 Box plots of total pre-hospital delay and decision time**

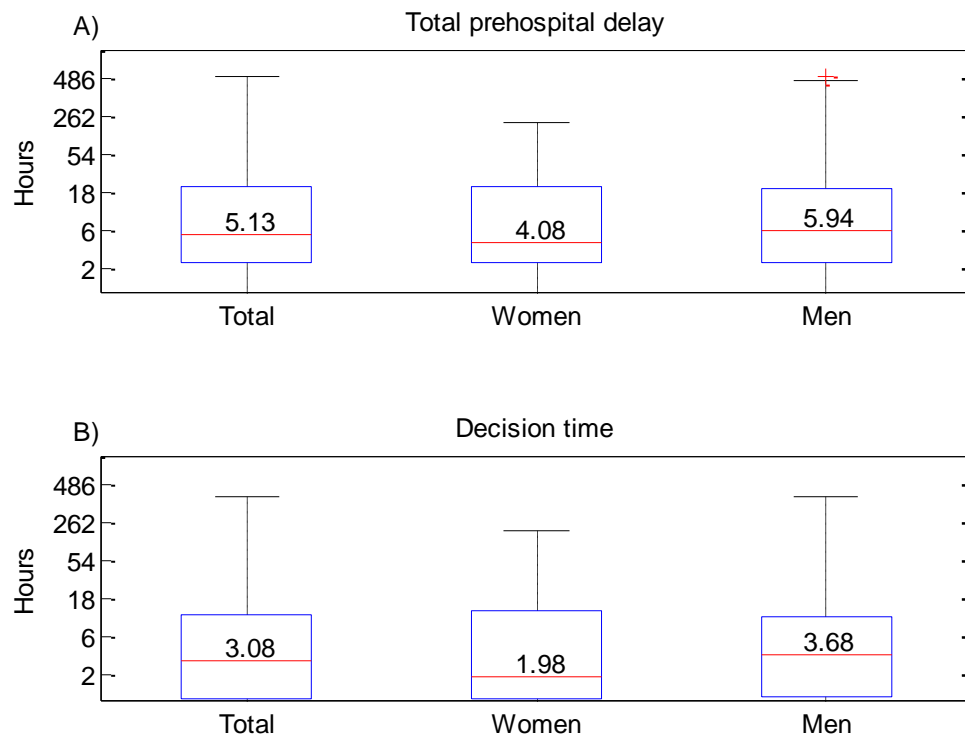

Supplement: Additional file 1: — Box plots of total pre-hospital delay and decision time. (PDF 171 kb) [file 12872_2016_271_MOESM1_ESM.pdf]
